# Soft X-ray imaging with coherence tomography in the water window spectral range using high-harmonic generation

**DOI:** 10.1038/s41377-025-02057-9

**Published:** 2026-01-22

**Authors:** Julius Reinhard, Felix Wiesner, Martin Hennecke, Themistoklis Sidiropoulos, Sophia Kaleta, Julian Späthe, Johann Jakob Abel, Martin Wünsche, Gabriele Schmidl, Jonathan Plentz, Uwe Hübner, Katharina Freiberg, Jonathan Apell, Stephanie Lippmann, Matthias Schnürer, Stefan Eisebitt, Gerhard G. Paulus, Silvio Fuchs

**Affiliations:** 1 Institute of Optics and Quantum Electronics, Friedrich Schiller University Jena, Jena, Germany; 2 Helmholtz Institute Jena, GSI Helmholtzzentrum für Schwerionenforschung, Jena, Germany; 3 Max-Born-Institut für Nichtlineare Optik und Kurzzeitspektroskopie, Berlin, Germany; 4 Indigo Optical Systems GmbH, Jena, Germany; 5 Leibniz Institute of Photonic Technology (Leibniz-IPHT), Jena, Germany; 6 Otto Schott Institute of Materials Research Friedrich Schiller University Jena, Jena, Germany; 7 Institute of Materials Science and Engineering, Chemnitz University of Technology, Chemnitz, Germany; 8 Institute of Applied Physics, Friedrich Schiller University Jena, Jena, Germany; 9 Institute for Optics and Atomic Physics, Technische Universität Berlin, Berlin, Germany; 10 Laserinstitut Hochschule Mittweida, University of Applied Science Mittweida, Mittweida, Germany

**Keywords:** High-harmonic generation, X-rays, Imaging and sensing, Optical metrology

## Abstract

High-harmonic generation (HHG) is used as a source for various imaging applications in the extreme ultraviolet spectral range. It offers spatially coherent radiation and unique elemental contrast with the potential for attosecond time resolution. The unfavorable efficiency scaling to higher photon energies prevented the imaging application in the soft X-ray range so far. In this work we demonstrate the feasibility of using harmonics for imaging in the water window spectral region (284 eV to 532 eV). We achieve nondestructive depth profile imaging in a heterostructure by utilizing a broadband and noise-resistant technique called soft X-ray Coherence Tomography (SXCT) at a high-flux lab-scale HHG source. SXCT is derived from Optical Coherence Tomography, a Fourier based technique that can use the full bandwidth of the source to reach an axial resolution of 12 nm in this demonstration. The employed source covers the entire water window, with a photon flux exceeding 10^6^ photons/eV/s at a photon energy of 500 eV. We show local cross sections of a sample consisting of Aluminium oxide and Platinum layers of varying thickness on a Zinc oxide substrate. We validate the findings with scanning and transmission electron microscopy after preparation with focused ion beam milling.

## Abstract (web summary)

Water window imaging with high-harmonic generation is achieved using soft X-ray coherence tomography (SXCT), revealing the cross-sectional structure of buried nanolayers with 12 nm axial resolution.

## Graphical abstract

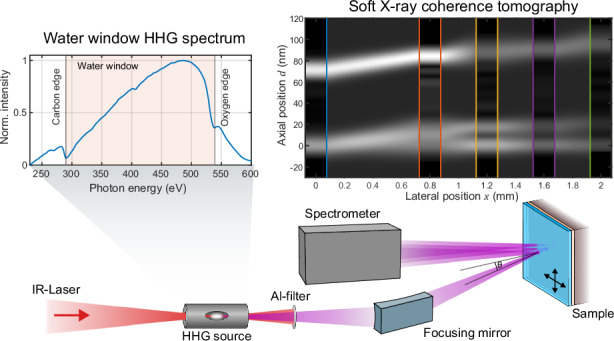

## Introduction

**Fig. 1:**
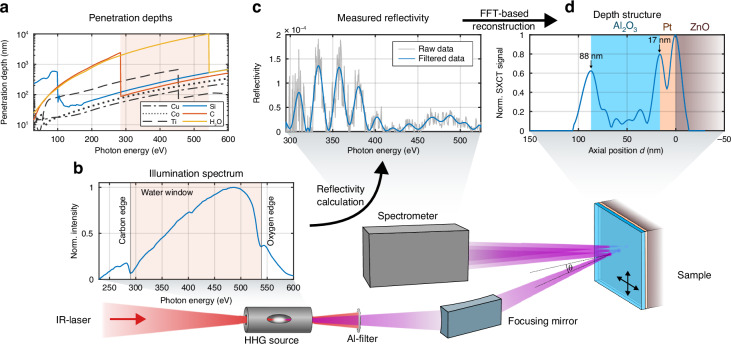
**Attenuation lengths of various materials, as well as illumination spectrum, measured sample reflectivity, and reconstructed depth structure are arranged on top of a schematic representation of the setup**. **a** The attenuation length, or penetration depth, describes the distance over which light of a certain energy can propagate before its intensity drops to 1/e $\approx$ 37%. In the EUV range between 30 eV and 100 eV, most materials have an attenuation length of a few tens of nanometers at most, except for silicon (blue). The water window is defined by the absorption edges of carbon (orange) and oxygen (see H_2_O, yellow), and allows penetration of up to 10 µm in water, as implied by the name. Compared to the EUV range, a significantly greater penetration depth of $>$100 nm is also achieved for almost all other materials, such as the metals selected as examples. **b** The illumination spectrum spans the entire water window spectral range. It is generated with high-harmonic generation driven by a few-cycle 2.1 µm laser. The spectrum is shaped by aluminium absorption filters, which contain oxides as well as carbon deposits. These features result in the strongly pronounced absorption edges of oxygen and carbon, which define the water window boundaries. **c** The SXR light is focused onto the sample with a toroidal mirror. It is reflected at the various interfaces within the sample, leading to intensity modulations in the reflected spectrum due to interference. The high roughness of the sample leads to a significant reduction in reflectivity, resulting in a very noisy raw signal (grey). The signal relevant for the reconstruction was filtered out using a low-pass Fourier filter (blue). This data corresponds to the scan position at $x$ = 1.2 mm from the linescan shown in Fig. 2 (yellow curve). **d** The depth structure of the sample was retrieved using the Fourier based SXCT algorithm. Three interfaces are reconstructed, which correspond to two layers with a thickness of 71 nm and 17 nm respectively on top of the substrate. By scanning across the sample, additional lateral information can be obtained

High-harmonic generation (HHG) has proven to be an exceptional source for nanoscale extreme ultraviolet (EUV) imaging due to its laser-like properties and small laboratory footprint^1–3^. In particular, coherent imaging methods such as ptychography benefit from the high spatial coherence and have demonstrated their remarkable potential in recent years achieving wavelength-scale resolution^4–8^. In addition, the strong elemental contrast opens up a wide field of applications from semiconductor studies^9^ and mask inspection^10^ to biological samples^11–13^. Even buried structures can be examined using EUV light from HHG sources^14–16^. However, the penetration depth is severely limited by the strong absorption of most materials, which is the range of a few ten nanometers (Fig. 1a).

Further decreasing the wavelength to the soft X-ray (SXR) range would not only decisively increase the penetration depth to a few hundred nanometers for a great variety of materials, but would also enhance the resolution as demonstrated at synchrotron sources^17^. The so-called water window is of particular interest. It is defined by the absorption edges of carbon (≈ 284 eV or 4.4 nm) and oxygen (≈ 532 eV or 2.3 nm), and within this spectral range, penetration depths of up to 10 µm are achievable in water (see Fig. 1a). This window is especially valued for its inherent contrast when imaging biological samples^18–20^. Moreover, due to the ubiquitous presence of carbon and oxygen in many materials, it serves as a key transmission window for a wide range of investigations in the SXR regime. Penetration depths on the order of several hundred nanometers are typical in this spectral region, which is significantly greater than those achievable in the EUV range or by electron microscopy.

HHG-driven imaging in the soft X-ray or, more specifically, the water window spectral range has not yet been demonstrated, primarily for two reasons. On the one hand, the available photon flux of HHG sources in the SXR range is severely limited. Extending the HHG spectral range into the water window typically requires ultrafast driver lasers with wavelengths above 1 µm. Unfortunately, this reduces the conversion efficiency of the process, which scales with ∼*λ*^–5^ to *λ*^–6^ (ref. ^21^). Therefore, SXR-HHG have only been used for various spectroscopic applications that exploit the femto- or even attosecond pulse lengths^22–24^. On the other hand, the intrinsically broadband harmonic spectrum is not efficiently used by the established EUV imaging methods like Ptychography, which typically require monochromatic light and consequently only use a small fraction of the available photon flux. Thus, intrinsically broadband imaging techniques are better suited for flux-limited HHG sources in the water window spectral range.

In this work, we demonstrate the feasibility of HHG-driven imaging in the water window on solid-state nanostructures by combining a cutting-edge WW-HHG source with a broadband and noise resistant technique called soft X-ray coherence tomography (SXCT). The HHG source is driven by a few-cycle 2.1 µm laser^25^ and generates a broad and continuous spectrum covering the entire water window with more than 5⋅10^5^ ph/eV/s at the oxygen K-edge (2.33 nm)^26^. To our knowledge, no other HHG source was able to apply these photon numbers in experiments yet. Moreover, the broadband acquisition of a wide spectral region enables the simultaneous recording of highly correlated spectral features along atomic resonances and diffraction peaks in a very time-efficient manner. This unique capabilities have recently been demonstrated in time-resolved spectroscopy^27^ and diffraction^28^ experiments. In this work, SXCT exploits the full bandwidth of the source to reconstruct depth structures with nanometer-scale axial resolution. This imaging technique is derived from spectral-domain optical coherence tomography (OCT^29^) and functions as a reflective method, enabling the investigation of structures on bulk materials such as microchips. It even enables the study of weakly reflective samples due to its Fourier-based nature, which provides exceptional noise resistance. Furthermore, three-dimensional information can be obtained by laterally scanning the sample.

We investigated a sample composed of an aluminum oxide layer and a platinum layer deposited on a zinc oxide substrate using SXCT. A cross-sectional image was obtained scanning across the edge of the buried platinum layer, achieving a depth resolution of 12 nm. To validate the axial structure and inhomogeneities detected with SXCT, we conducted scanning and transmission electron microscopy (SEM and TEM) following focused ion beam (FIB) milling.

## Results

### HHG source and SXR coherence tomography

The demonstration of HHG-driven water window imaging was enabled by a high-flux HHG source covering the entire water window spectral range^25,26^. The source is based on an optical parametric chirped-pulse amplification (OPCPA) system, driven by a 500 W thin-disk laser to reach a central wavelength of 2.1 µm and an average power of 28 W at pulse duration of 27 fs. The beam is focused with a 750 mm lens into a helium gas cell, where the HHG process occurs. The resulting spectrum covers a broad spectral range from 200 eV to 600 eV, with a photon flux of 5⋅10^5^ ph/eV/s at the oxygen K-edge at ≈ 532 eV^26^. The HHG spectrum, which was used for the SXCT measurements is shown in Fig. 1b.

Soft X-ray coherence tomography (SXCT) extends optical coherence tomography (OCT) to shorter wavelengths and broader spectral ranges, enabling high-resolution imaging in the soft X-ray regime. The axial resolution Δ*d* is independent of the focusing geometry. Instead, it is defined by the coherence length ${l}_{c}={\lambda }^{2}/\Delta \lambda$, i.e. a shorter wavelength and broader spectrum lead to higher axial resolution. With commercial OCT systems working in the infrared spectral range, axial resolutions of a few micrometers are achieved^30^. In recent years, EUV coherence tomography (XCT), the extreme ultraviolet descendant of OCT, was established in the silicon transmission window between 30 eV and 100 eV^15,31–33^. This enabled an axial resolution of ≈15 nm, which has been demonstrated in a laboratory setup using HHG^34^.

SXCT is implemented as a common-path Fourier-domain OCT variant^14,35^, in which the sample’s spectral response is detected by a spectrometer without the use of a beam splitter. Instead, the separation of the illumination and reflected beam is achieved through an oblique incidence angle. Consequently, the axial resolution $\Delta d$ additionally depends on the incidence angle $\theta$ relative to the surface normal, following $\Delta d={\lambda }^{2}/(\Delta \lambda \cos \theta )$. In order to enhance the reflectivity and thereby increase the signal strength, particularly at short wavelengths, larger incidence angles can be utilized. However, this results in a slightly reduced axial resolution. A detailed discussion of this trade-off is found in the methods section. In this experiment, we used an incidence angle of *θ* = 72°. Consequently, a theoretical maximum resolution of 10.6 nm can be achieved, assuming a rectangular spectral range of 295 eV to 524 eV and the absence of a filter function to suppress Fourier artifacts.

The setup is schematically depicted in Fig. 1. A toroidal mirror focuses the SXR light from the HHG source onto the sample, forming an elliptical spot of 150 µm × 30 µm due to *θ* = 72°. The light reflected from internal sample structures interferes with the light reflected from the surface, which serves as a reference in the common path SXCT scheme. This interference induces modulations in the spectrum of the reflected light, which are detected by a grating spectrometer. By measuring the incident spectrum (Fig. 1b), the sample reflectivity (Fig. 1c) can be determined.

The sample’s depth structure in the illuminated region is encoded in the spectral modulations of this reflectivity and can be reconstructed using a Fourier-based algorithm. The Fourier transform of the intensity reflectivity of the sample represents the autocorrelation of the axial sample structure. In contrast to typical OCT systems with one dominant surface reflection, all interfaces in our sample exhibit reflectivities of a similar magnitude. This leads to an intrinsically ambiguous autocorrelation, which contains not only the true interface positions but also spurious auto-correlation peaks arising from the interference between different buried interfaces. However, the unambiguous axial structure can be reconstructed from the autocorrelation by employing a phase retrieval algorithm. In this work, we use a three-step one-dimensional phase retrieval algorithm originally developed for XCT, which is described in detail in the supplementary information in Fuchs et al^15^.

Due to the Fourier-based nature of the method, SXCT is highly resistant to noise, similar to the principle of lock-in amplifiers. Individual interfaces within the sample contribute to distinct modulation frequencies in the spectrum. Thus, broad-bandwidth noise can be efficiently filtered out, resulting in a high signal-to-noise ratio, as demonstrated in Fig. 1c. The measured reflectivity of one sample position is shown in gray, while the reflectivity after low-pass Fourier filtering is shown in blue. Using this processed reflectivity as input, the established XCT reconstruction algorithm^15^ extracts the depth profile (Fig. 1d). Each peak corresponds to an interface in the sample, where the reflections occur.

A cross-sectional or three-dimensional image of the sample can be obtained by laterally scanning in one or two dimensions while retrieving the axial structure point by point. As a consequence, the lateral resolution is independent of the axial resolution and defined by the size of the SXR probe on the sample which in our case was 150 µm × 30 µm.

### SXCT cross section

**Fig. 2:**
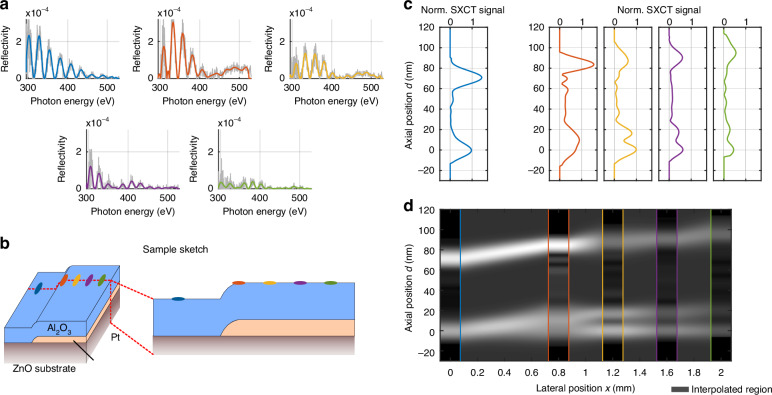
**Cross-sectional imaging using SXCT**. **a** We performed a linescan, measuring reflectivities at five different positions of the investigated sample. It is depicted schematically in (**b**). It consists of a thin Al_2_O_3_ layer on a ZnO substrate, with a buried Pt layer present in a specific region of the sample. The five scan positions are highlighted in different colors. Without Pt-layer (blue) a reflectivity of up to 2$\cdot$10^−4^ was measured, strongly decreasing at higher energies. At positions with Pt-layer an additional modulation frequency is observed in the spectra. Furthermore, the total signal decreases with increasing distance from the platinum edge (yellow to green), indicating an increasing roughness. **c** Using the SXCT algorithm^15^ the depth structure of the sample can be reconstructed for each scan position. The Al_2_O_3_ layer can be resolved throughout the sample with a varying thickness between 71 nm and 75 nm. In addition, the thinner buried Pt layer behind the edge can also be resolved with increasing thickness between 17 nm and 20 nm. At the second position (orange), the comparatively large SXR focus averages over a varying Pt thickness, leading to a broadened signal. **d** A cross-section was generated from the five depth profiles by interpolating the regions between the scan positions

We performed SXCT on a layered test sample, consisting of a ≈70 nm thick Al_2_O_3_ layer on a ZnO substrate. In a specific region of the sample, an additional Pt layer is buried below the Al_2_O_3_ with a thickness of ≈20 nm. A linescan (a so-called B-scan in OCT terminology) was conducted with five different lateral points (A-scan), each having an exposure time of 15 min. A schematic depiction of the sample highlighting the scan positions in different colors is shown in Fig. 2b.

The measured reflectivities of each lateral sample point are shown in Fig. 2a. The first scan position without Pt layer (blue) shows a modulation with a maximum reflectivity of 2⋅10^−4^, which decreases with increasing photon energy. This value is an order of magnitude lower than expected based on theoretical data^36^. The deviations can be attributed to differences in material density and, more importantly, to non-negligible interface roughness. The subsequent scan positions show an additional modulation frequency generated by the Pt layer. At the second scan position (orange), a slight increase in reflectivity is observed, particularly at higher photon energies. However, as the scan progresses (yellow to green), the overall signal decreases with increasing distance from the platinum edge.

The depth profiles for each scan position were reconstructed from the measured reflectivities using the SXCT algorithm which applies an one dimensional phase retrieval (Fig. 2c)^15^. The depicted normalized SXCT signal is the phase-retrieved absolute value of the depth-dependent field reflectivity. For a meaningful comparison across the linescan, all profiles in Fig. 2c were normalized using a single global factor. This factor was set by the amplitude of the Pt/ZnO substrate interface peak (*d* = 0) from the measurement shown in Fig. 1d, which corresponds to scan position 3 (yellow curve). This approach preserves the relative signal amplitudes between all measurements. The graphs were aligned such that the deepest interface - the substrate surface—is located at *d* = 0 nm. From these profiles, a cross section was generated by interpolating between the scan positions (Fig. 2d). The individual depth plots are positioned above the cross-section according to their respective lateral coordinate $x$. At the first scan position (blue curve) two peaks are visible: the Al_2_O_3_ surface (axial position *d* = 71 nm) and the Al_2_O_3_/ZnO interface (*d* = 0 nm). A shift in the surface position becomes apparent for the following scan positions. As the lateral position $x$ increases, a shift of the surface position becomes apparent. In addition, a broadening of the signal at the Al_2_O_3_/Pt interface (*d* = 15 nm) becomes visible at *x* = 0.8 mm (orange). This is due to the presence of the buried Pt layer, which apparently does not have a sharp lateral edge, but has a gradually increasing thickness within the illumination spot. This is supported by the reduced modulation depth and irregular modulation frequency observed in Fig. 2a (orange). At *x* = 1.2 mm (yellow) the front (*d* = 17 nm) and back (*d* = 0 nm) of the Pt layer are clearly distinguishable indicating a resolution of at least 17 nm. The Pt thickness further increases at *x* = 1.6 mm (purple) and *x* = 2 mm (green) reaching 21 nm. The thickness of the Al_2_O_3_ layer increases as well to 75 nm.

Additionally, the ratio between the Al_2_O_3_/Pt interface peak (*d* ≈ 20 nm) and the Pt/ZnO interface peak (*d* = 0 nm) decreases from *x* = 1.2 mm (yellow) to *x* = 2 mm (green). Combined with the decrease in overall reflectivity observed in Fig. 2a, this indicates that the roughness of the platinum layer increases with its thickness.

The peak positions were determined by Gaussian fitting of the depth structure. This also revealed a FWHM of the Al_2_O_3_/Pt interface at the third scanning position (yellow, *d* = 17 nm) of 12 nm, which defines the achieved resolution. For the purple (*x* = 1.6 mm) and green (*x* = 2 mm) scanning positions, the width increased to 15 nm, indicating a thickness variation of the Pt layer of ±1.5 nm in the illuminated region.

### Validation with electron microscopy

**Fig. 3:**
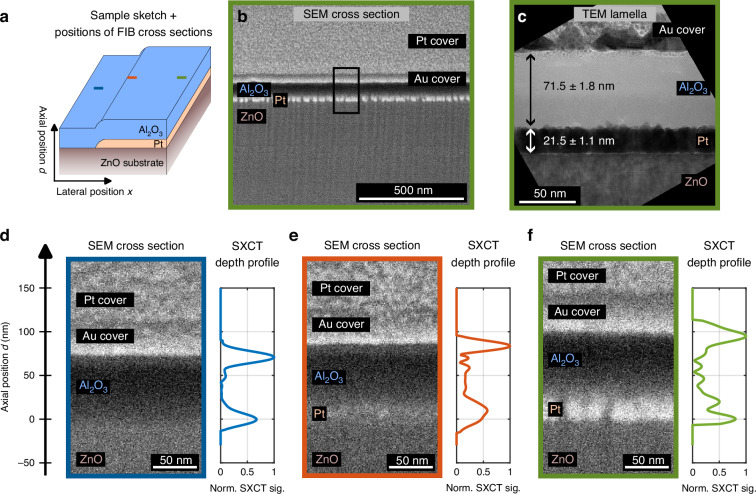
**Validation of SXCT results with electron microscopy**. **a** Three regions on the sample are distinguished: Al_2_O_3_ on ZnO substrate (blue position), Al_2_O_3_ on Pt on ZnO substrate (green position) and the region in between with the Pt layer slowly forming (orange position). At each position a FIB cross section was made and compared to SXCT depth profiles. **b** A SEM image of a FIB cross section at the green position, shows the Al_2_O_3_ and Pt layers, as well as the ZnO substrate and additional gold and platinum layers deposited in preparation for FIB milling. The bright Pt layer is easily recognizable and its high granularity and roughness is revealed. **c** Section of the TEM lamella, prepared from the cross section shown in b. The Al_2_O_3_ layer is visible on top of the inhomogeneous and rough Pt layer. Averaging over the full width of 5 µm, axial distances of 71.5 nm and 21.5 nm could be confirmed for Al_2_O_3_ and Pt, respectively. **d**–**f** FIB cross sections were made at different sample positions and compared to corresponding SXCT depth profiles. The positions of the interfaces are highly consistent. It is confirmed that the thickness of the Pt layer gradually increases, rather than having a sharp edge

We verified the SXCT results by performing scanning and transmission electron microscopy (SEM/TEM) investigations of the sample after FIB preparation. We compared three positions with corresponding SXCT scan positions: Al_2_O_3_ on substrate (blue position and blue frame in Fig. 3d), Al_2_O_3_ with buried Pt (green, Fig. 3f) and close to the edge (orange, Fig. 3e). For all three positions, we prepared FIB cross sections and imaged them using SEM. In addition, we extracted a thin lamella with a width of ≈5 µm from the Al_2_O_3_/Pt position (green) and investigated it with TEM (Fig. 3c). To protect the sample surface during FIB preparation, the sample was coated with gold and platinum after SXCT measurements. Details can be found in the Methods section.

A large field of view from the FIB cross section at the green position is presented in Fig. 3b. It can be clearly seen from the granular structure that the platinum grows in columns and does not form a homogeneous layer. As a result, the layer exhibits a high roughness. On top of this, the Al_2_O_3_/Au interface appears blurred (see also the enlarged area of the SEM image in Fig. 3f), indicating the transition of roughness from the buried Pt layer to the surface, which further reduces the reflectivity.

The reasons for columnar growth of platinum can be attributed to two main factors. On the one hand, these are the deposition conditions, specifically the deposition pressure (Ar pressure) and temperature of the substrate surface (Thornton’s model^37^). On the other hand, it is the growth of platinum on an oxide surface, which can be described as island growth (Volmer-Weber growth mode^38^). This combination results in a highly inhomogeneous vertical growth, particularly in the case of very thin layers. A TEM investigation at the same position (Fig. 3c) confirms the non-negligible roughness of the Pt layer.

The lower part of Fig. 3 shows SEM images at three different lateral positions, enabling a direct comparison with the corresponding SXCT depth profiles. The SEM images were aligned, such that the ZnO/Al_2_O_3_ or ZnO/Pt interface is at *d* = 0. As expected, no Pt is visible in the blue position (Fig. 3d) and a sharp interface between the dark Al_2_O_3_ and the bright gold cover is observed at *d* = 71 nm, which is in good agreement with the SXCT results. At the orange position (Fig. 3e), the emergence of the Pt layer on top of the ZnO is visible in both the SEM image and the SXCT profile. The SXCT depth profile however, is integrated over a varying layer thickness due to the lateral extent of the SXR probe (150 µm×300 µm). For this reason, the interfaces cannot be resolved in this transition region of increasing Pt thickness. The enlarged image of the green position (Fig. 3f), away from the boundary region, shows the fully formed Pt layer. The positions of the front and back of the Pt layer are in good agreement for SEM and SXCT.

From a series of ten measurements along the width of the TEM lamella (Fig. 3c), a thickness of 21.5±1.1 nm was determined for the platinum layer and 71.5±1.8 nm for the Al_2_O_3_ layer. It is important to note that the error reflects the variation of the layer thickness across the width of the TEM lamella, rather than the method’s inaccuracy.

## Discussion

In this work, we showed that imaging with high-harmonic generation (HHG) sources is feasible in the water window (WW) spectral range. This was achieved by combining an HHG source that spans the whole WW range up to the oxygen edge and the flux efficient and noise resistant method of soft X-ray coherence tomography (SXCT). We imaged the internal sample structure of a layer system consisting of Al_2_O_3_ and Pt layers with an axial resolution of 12 nm, which is close to the theoretical limit of 10 nm for this spectral range. The results of non-destructive SXCT were in remarkable agreement with those obtained using established, but destructive, scanning and transmission electron microscopy (TEM), both in terms of absolute layer thickness (TEM: 21.5 nm, SXCT: 21 nm) and thickness variation (TEM: 1.1 nm, SXCT: 1.5 nm) for the buried Pt layer. The thickness variation or high roughness and therefore low reflectivity of the test sample demonstrate the method’s applicability to real-world samples. For higher-reflectivity samples, even lower axial resolutions in the single-digit nanometer range can be achieved by using steeper angles of incidence. This was already demonstrated for a highly reflective periodic sample using an incoherent laser-plasma source, achieving an axial resolution of <5 nm^39^.

Overall, our results represent an important first step towards exploiting the potential of water window harmonics for non-destructive imaging. Soft X-ray coherence tomography (SXCT) offers a valuable approach for studying materials whose absorption is too strong in the extreme ultraviolet (EUV) range. Simultaneously, it provides strong elemental contrast, even for low-Z materials, a common challenge encountered in conventional TEM methodologies. In the EUV range, XCT has already been applied to material identification as well as the reconstruction of roughness and layer thicknesses well below the resolution limit^31^. This approach can be extended into the SXR range, enabling further advancements. Cross-sectional imaging with HHG radiation will also facilitate the study of dynamic effects in buried layers with high spatial and femto- or even attosecond temporal resolution^28^. To achieve higher lateral resolution, the spot size could be reduced to sub-micrometer levels using more sophisticated demagnifying optics. Additionally, ongoing advancements in ptychographic algorithms aim to leverage broad spectra more effectively^40^. Together with the development of even more powerful HHG sources, these innovations pave the way for future applications in the water window.

## Materials and methods

### Experimental setup

The soft X-ray spectra were acquired using a combined $\theta -2\theta$ reflectometry and spectroscopy setup, enabling transmission and reflection geometries for $\theta$ angles of ${45}^{\circ }-{90}^{\circ }$. The soft X-rays were provided by a laboratory light source based on high-harmonic generation, operated with the noble gas helium (≈2.2 bar gas pressure). The HHG process was driven by 2.1 µm, 27 fs (FWHM) infrared pulses generated via optical parametric chirped-pulse amplification (OPCPA). The OPCPA system features a monolithic concept, deriving the seed and pump pulses for the OPA stages from the same 500 W thin-disk pump laser (TRUMPF Scientific Lasers Dira 500-10), using a 2.1 µm front-end (Fastlite) for signal generation^25,26^. The system delivers pulses with 10 kHz repetition rate at an average power of 28 W, which are focused into a helium gas cell to generate p-polarized ≤27 fs soft X-ray pulses covering a continuous spectrum from 200 to 600 eV. The resulting photon flux is ≈5⋅10^5^ph/eV/s @ O K-edge, ≈10^6^ph/eV/s @ Ti L-edge and ≈6⋅10^6^ph/eV/s @ C K-edge, determined using a grating- and CCD-based spectrometer with absolute sensitivity calibration by the German metrology institute (PTB). The broadband pulses are focused onto the sample upon reflection by a toroidal mirror, leading to a footprint of the focal spot of 150 × 300 µm^2^ (FWHM) for an angle of 72° to the surface normal. After reflection by the sample, the spectrum is dispersed by a reflection variable line spacing (VLS) grating (Hitachi 001-0450, 2400 l/mm central line density) and recorded on a CCD camera (Greateyes GE 2048 512 BI).

### Data analysis

**Fig. 4:**
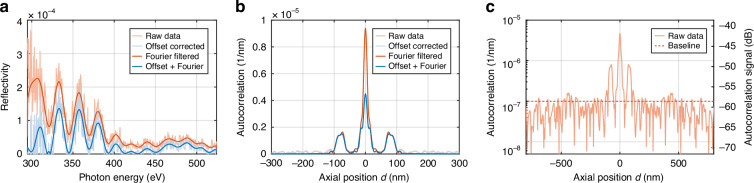
**Denoising and offset correction of measured reflectivity data for reconstruction and sensitivity analysis**. **a** The reflectivity of the sample at scan position 3 ($x$ = 1.2 mm) is calculated by dividing the reflected spectrum of the sample by the illumination spectrum. The raw reflectivity gained this way is shown in light orange. To denoise the data and eliminate high frequency modulations, a Fourier filter is applied, i.e. all values above or below $\pm$120 nm in the spatial domain are set to zero (orange curve). Additionally, a background correction is applied, by subtracting a constant offset in the wavelength domain (light blue). For SXCT reconstruction both filters are combined (blue). **b** Autocorrelation of the sample structure prior to phase reconstruction. The offset correction reduces the signal at zero depth in the autocorrelation to ensure convergence of the phase retrieval algorithm. **c** Same autocorrelation signal of the unfiltered spectrum as in (**b**) but on a logarithmic scale and dB-scale. 0 dB corresponds to a mirror with reflectivity $R$=1. The dB-scale directly indicates the absolute reflectivity by peak height

A reference spectrum was recorded prior to the linescan by moving the sample out of the beam path and aligning the spectrometer to the transmitted radiation. Calibration was done by fitting the grating equation to the carbon, nitrogen and oxygen edges visible in the spectra. We then applied offset correction, interpolation into the photon energy domain, and denoising to the recorded spectra, as explained below. The impact of these processing steps on the reflectivity and sample autocorrelation is shown in Fig. 4.

The low reflectivity and incoherently scattered radiation from the inhomogeneous sample cause a significant offset in the measured reflectivity. In the sample autocorrelation this corresponds to an increased signal at zero depth. The phase retrieval relies on the correct correlation between the different features in the sample’s autocorrelation. Therefore, an offset in the sample reflectivity prevents convergence of the phase retrieval. The effects of the offset correction on the sample reflectivity and autocorrelation are shown in Fig. 4a, b. In this work, we assume a constant offset on the camera, i.e. in wavelength. We find that applying an offset subtraction of 0.27 Cts/s yields good results for all points of the linescan except for the point directly at the transition between Pt and Al_2_O_3_, where a significantly larger offset is observed and a correction by 0.54 Cts/s is needed. The reason for this is the laterally inhomogeneous sample within the focal spot. The reflectivity is averaged over parts with a varying thickness of the Pt layer.

Due to the nonlinear relation between wavelength and photon energy $\lambda \propto 1/E$ the varying energy width of the camera pixels needs to be corrected by dividing by the energy spacing of the grid $\Delta E$. The sample reflectivity is obtained by dividing the reflected spectrum of the sample by the reference spectrum.

Figure 4c shows the autocorrelation of the raw, unfiltered reflectivity measured at *x* = 1.2 mm, plotted on a logarithmic scale. The dB values directly represent reflectivity, with 0 dB corresponding to an ideal mirror ($R=1$). The sensitivity of the measurement can be estimated by evaluating the noise floor of the signal, which was found to be 58 dB.

The interpolation of the SXCT cross section (Fig. 2b) was based on Gaussian curve fitting. For each lateral position Gaussian curves were fitted to the peaks corresponding to the interface positions. The parameters of the curves (position, width and amplitude) were linearly interpolated between the lateral positions and used to plot the cross-section. For the second scan position (orange, $x$ = 0.8 mm) three Gaussians were fitted to describe the broadened peak between $d$ = -10 nm and $d$ = 20 nm. One Gaussian corresponds to the substrate surface at $d$ = 0 nm, while the other two are used to describe the varying thickness of the Pt layer. The mean value of these two positions is considered as the average position of the Pt/Al_2_O_3_ interface at $x$ = 0.8 mm.

### Characteristic properties of the SXCT setup

**Fig. 5:**
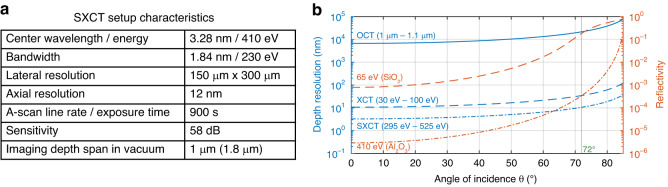
**Setup characteristics and resolution/reflectivity trade off diagram**. **a** The characteristics of the SXCT setup are summarized in a comprehensive table. Due to the nonlinear relationship between wavelength and photon energy, the center wavelength does not correspond to the center energy—only the spectral boundaries do. The lateral resolution is defined by the focal spot size. The axial resolution was determined using a Gaussian fit to the reconstructed depth structure. The sensitivity was determined on basis of Fig. 4c. The imaging depth span is defined by the spectrometer resolution, which was estimated from the illumination spectrum or can be calculated based on the pixelation at the highest photon energy used (in brackets). **b** The diagram illustrates the trade-off between axial resolution (blue) and reflectivity (orange) as a function of the angle of incidence $\theta$. The theoretical resolution was calculated assuming a rectangular input spectrum. Typical commercial OCT systems achieve axial resolutions of a few micrometers at normal incidence. XCT, the EUV predecessor of SXCT, typically employs a spectral bandwidth of 70 eV, theoretically enabling resolutions of approximately 10 nm at normal incidence. In practice, XCT is often operated at an incidence angle of 15°, which only slightly reduces the resolution. SXCT, in contrast, uses a broader bandwidth of 230 eV, theoretically allowing axial resolutions of around 3 nm at normal incidence. However, due to low reflectivities in this spectral range, a significantly higher angle of incidence of 72° was chosen in our experiment, resulting in a theoretical resolution limit of approximately 10 nm. The reflectivity curves were calculated for representative materials at the central energies of XCT (65 eV, SiO_2_) and SXCT (410 eV, Al_2_O_3_) based on data of Henke et al.^36^

The most important parameters of the presented experimental implementation of water window SXCT with high harmonics are summarized in Fig. 5a.

The specified central energy (410 eV) and wavelength (3.28 nm) do not correspond exactly due to their non-linear relationship, but both lie in the center of the specified bandwidths (1.84 nm and 230 eV, respectively). The lateral resolution of 150 × 300 µm^2^ is defined by the focal spot size, whereas the experimentally achieved axial resolution of 12 nm was determined using a Gaussian fit to the reconstructed depth structure of scan position 3. Using steeper angles of incidence an axial resolution of down to ≈nm is achievable^39^. The lateral resolution can be improved to a few hundred nanometers using more sophisticated demagnifying optics, as demonstrated with a HHG source^41^, and even down to 20 nm at a synchrotron light source^42^.

The A-scan line rate, or rather the exposure time for each position, was 900 s. However, this time is not inherent to the system or the method, but was necessitated by the comparatively low sample reflectivity and limited flux. Smoother samples or even flatter angles can reduce the exposure time, as can future HHG sources with even higher flux.

The sensitivity is drawn from the noise levels in Fig. 4c). Shot noise and scattered light from the grating are the most prominent noise sources and limit the achieved sensitivity to 58 dB compared to a mirror with a reflectivity of $R$=1. However, this could be improved by better gratings and higher photon flux.

The imaging depth span in vacuum describes the maximum depth at which a structure can still be imaged by the spectrometer. This limitation arises because deeper layers induce higher-frequency modulations in the measured reflectivity spectrum. Consequently, the spectrometer resolution imposes a limit on the maximum detectable modulation frequency—and therefore on the maximum accessible depth. In our case, the detector pixelation leads to an energy discretization of 0.23 eV at 295 eV and 0.56 eV at 525 eV. At an incidence angle of 72°, this corresponds to maximum detectable depths of 4.3 µm and 1.8 µm, respectively. In practice, however, the resolution is further limited by the finite source/focus size and grating aberrations. Based on the absorption edges in the illumination spectrum (see Fig. 1b), we estimate the effective resolution to be approximately 2 eV, corresponding to a practical depth limit of around 1 µm. If needed, the spectral resolution—and thus the imaging depth—could be improved relatively easily by employing a grating with higher dispersion.

It should be noted that the specifications provided in Fig. 5a) refer to the current state of a first proof-of-concept experiment and do not reflect the full potential or any fundamental limit of the method. With further development, significant improvements in performance can be expected as described.

### Resolution-reflectivity trade-off

Due to the unexpectedly low reflectivity of the sample, we chose a shallow incidence angle of 72°, accepting a reduction in depth resolution as a trade-off. This relationship is illustrated in detail in Fig. 5b, which shows the maximum theoretical resolution for a rectangular input spectrum of defined bandwidth, without applying additional filter functions.

In general, higher depth resolution in coherence tomography is achieved with broader spectral bandwidths. For instance, commercial OCT systems operating with a bandwidth of 0.11 eV (corresponding to 1 µm to 1.1 µm) typically reach axial resolutions of a few micrometers at normal incidence. In contrast, XCT—the EUV predecessor of SXCT—operates with a bandwidth of 70 eV (30–100 eV), enabling resolutions down to 10 nm. With the spectral range used in this work (295–525 eV), SXCT can in principle achieve axial resolutions of down to 3 nm. Due to projection effects, this resolution decreases with increasing angle $\theta$, dropping by approximately one order of magnitude at extremely shallow angles beyond 80°.

The reflectivity as a function of incidence angle was calculated based on data from Henke et al.^36^ for representative photon energies and materials—65 eV for SiO_2_ (XCT) and 410 eV for Al_2_O_3_ (SXCT)—assuming perfectly smooth surfaces. At 410 eV, the reflectivity at normal incidence is as low as 2$\cdot$10^−6^, but increases to nearly total reflection at grazing incidence. While lower reflectivity can be partly compensated by longer exposure times, the loss in axial resolution at shallow angles is irreversible. Therefore, optimizing this trade-off is essential when conducting an SXCT experiment.

### Electron microscopy

Cross sections were prepared using focused ion beam (FIB) milling in an FEI Helios Nanolab 600i dual-beam scanning electron microscope. The sample surface was protected by deposition of protective Au and Pt layers prior to FIB preparation. Cross sections were milled using an ion beam operating at 30 kV and 2.5 nA. SEM images were acquired using a secondary electron detector.

Cross section lamellae for transmission electron microscopy (TEM) were prepared using FIB and transferred to a Cu grid before applying the final thinning. Ion beam acceleration voltage and beam current were reduced down to 5 kV and 15 pA using stage tilts of 50.5° to 53.5°. TEM bright-field imaging was performed using a JEOL NEOARM 200 F instrument.

### Sample preparation

The platinum (Pt) and aluminium oxide (Al_2_O_3_) layers were deposited on a 10 × 10 mm^2^ and 1 mm thick ZnO substrate. The Pt coating was deposited using a sputter coater with Ar as the sputtering gas. The base pressure was 6 $\cdot$ 10^−2^mbar and the Ar pressure was 2 $\cdot$ 10^−1^mbar. A rate of approximately 0.14 nm/s, a voltage of 1.3 kV and a current of 14 mA were used.

The deposition of aluminium oxide (Al_2_O_3_) has been performed using the atomic layer deposition (ALD) at a temperature of 225 °C with 500 cycles. Each cycle took 70 ms. Trimethylaluminium (TMA) was the precursor for the vapor phase deposition.

To create two halves with different layer stacks on the substrate, a mask was used that was removed according to the coating sequence.

## Data Availability

The raw spectra used in this experiment are available via figshare (https://figshare.com/s/65e19afadb712934d49a) or via reasonable requests from the authors.
